# Protective Effects of Oxyberberine in 5-Fluorouracil-Induced Intestinal Mucositis in the Mice Model

**DOI:** 10.1155/2022/1238358

**Published:** 2022-05-30

**Authors:** Ronglei Huang, Gaoxiang Ai, Linjiang Zhong, Liting Mai, Jian-Nan Chen, Yuhong Liu, Yucui Li, Xiaoqi Huang, Ziren Su, Janis Ya-Xian Zhan

**Affiliations:** School of Pharmaceutical Sciences, Guangzhou University of Chinese Medicine, Guangzhou 510006, China

## Abstract

Berberine (BBR), a major active constituent of *Rhizoma coptidis*, was reported to exert beneficial effects on intestinal mucositis (IM) induced by 5-fluorouracil (5-FU). However, the bioavailability of BBR is extremely low, and its metabolites were perceived to contribute to its prominent pharmacological activities. Oxyberberine (OBB) is a gut metabolite of BBR, which has been reported to have a superior anti-inflammatory effect in experimental colitis. However, its anti-inflammatory effects against 5-FU-induced IM mice have not yet been investigated. Hence, the purpose of this study was to reveal the protective effects of OBB on IM induced by 5-FU and investigate its potential underlying mechanism. The IM mice model was induced by receiving 5-FU (60 mg/kg, i.p.) for five days. Meanwhile, BBR (50 mg/kg) and OBB (12.5, 25, and 50 mg/kg) were given prior to 30 min intraperitoneal injection of 5-FU for seven days. Results indicated that OBB ameliorated body weight loss, anorexia, diarrhea, and histopathological damage in 5-FU-induced IM mice. After OBB administration, the amounts of MDA, SOD, and GSH altered by IM were remarkably restored. OBB was also observed to dramatically decrease the levels of TNF-*α*, IL-8, IL-6, COX-2, and iNOS and promote the release of IL-10. Besides, OBB distinctly upregulated the mRNA expressions of PCNA, ZO-1, occludin, and mucin-1, which could improve intestinal homeostasis in IM mice. OBB also blocked the activation of the upstream TLR4/MyD88 signaling pathway, and then it inhibited the phosphorylation of the NF-*κ*B and MAPK pathways. Importantly, compared with BBR, OBB displayed a superior therapeutic effect to BBR in alleviating 5-FU-induced IM mice. These results indicated that OBB has considerable potential to become a novel candidate drug against IM.

## 1. Introduction

Intestinal mucositis (IM) is a recognized side effect of cancer chemotherapy [1]. 5-Fluorouracil (5-FU) is a frequently used chemotherapeutic drug in the treatment of malignant tumors [2], but can kill normal proliferating cells, especially the rapidly dividing intestinal mucosal cells [3]. Studies suggest that 50–80% of patients receiving 5-FU chemotherapy develop IM and the clinical manifestations are severe diarrhea, nausea, vomiting, and anorexia [4]. These side effects further increase the risk of systemic infection, seriously affect the prognosis of the disease, and even cause death [2]. Furthermore, the emergence of IM not only directly destroys the mucosal barrier but is also accompanied by the formation of oxygen-derived free radicals and the excessive secretion of proinflammatory cytokines [5]. Thus, drugs with antioxidant, anti-inflammatory properties, and a stable intestinal environment are considered beneficial to patients with mucositis [6]. However, so far, there is still lack of very effective drugs to treat IM. Therefore, there is an urgent need to develop new IM therapeutic drugs.

*Rhizoma coptidis* (RC), officially recorded in the *Chinese Pharmacopoeia*, has been commonly used to improve gastroenteritis for thousands of years in China [7]. Berberine (BBR), the most representative constituent of RC, is commonly used as an over-the-counter drug to treat diarrhea, colitis, and gastroenteritis [8]. In a recent study, BBR was reported to regulate fecal metabolites via modulating gut microbiota, thereby improving 5-FU-induced IM mice [9]. Increasing evidence has exhibited that BBR exerts multiple beneficial pharmacological activities including antidiarrhea, anti-inflammation, and antitumor [10]. However, the extremely low bioavailability of BBR is not enough to achieve its significant pharmacological activities [11]. This low blood concentration is thought to be caused by malabsorption and rapid metabolism [12]. Wang et al. studied that metabolites of BBR may contribute to its pharmacological effects [13]. BBR can be transformed into its metabolites through a variety of metabolic pathways, including dihydroberberine, berberine, demethylberberine, and jatrorrhizine. Several studies have indicated that the bioactivities (anticolitis, anti-inflammatory, antioxidant effects, etc.) of these metabolites were inferior to BBR [14]. Hence, it is worth exploring whether BBR is metabolized to produce active metabolites in the treatment of IM.

Our previous research found that oxidases in intestinal microbiota could oxidize BBR to its absorbable form, oxyberberine (OBB), which showed a higher absorption rate than BBR [8]. OBB has been revealed to exhibit superior anti-inflammatory [15], antifungal [16], antiarrhythmic [17], and hypoglycemic [18] effects than its prodrug BBR. Particularly, previous research studies demonstrated that OBB could regulate gastrointestinal function on DSS-induced colitis through blocking the TLR4-MyD88-NF-*κ*B signaling pathway, and its therapeutic effect is superior to BBR [8, 15]. Furthermore, compared with BBR, OBB exhibited excellent security [15]. Therefore, it is possible that OBB may be effective in therapy against IM. To the best of our knowledge, there is no study addressing the effect of OBB against 5-FU-induced IM. Consequently, we designed experiments to reveal the protective effects of OBB on IM induced by 5-FU and investigate its potential underlying mechanism.

## 2. Materials and Methods

### 2.1. Materials

OBB (purity *>*98%) was synthesized by our lab according to our previous methods [8]. BBR (purity >98%) was provided by Nanjing Guang Run Biotechnology Co., Ltd. (Nanjing, China). 5-FU (CAS:51-21-8) was bought from Sigma (St Louis, MO, USA). Sodium carboxymethyl cellulose (CMC-Na) was supplied by Sinopharm (Shanghai, China). GSH, SOD, MPO, and MDA assay kits were purchased from Nanjing Jiancheng Bioengineering Institute (Nanjing, China). The BCA protein kit was produced by Thermo Scientific Pierce (Guangzhou, China). ELISA kits (TNF-*α*, IL-6, IL-8, and IL-10) were purchased from Shanghai MLBIO Biotechnology Co., Ltd. (Shanghai, China). Primary antibodies against inhibitors of TLR4 (AF7017), MyD88 (DF6162), IRAK-1 (AF4742), p65 (AF5006), I*κ*B*α* (AF6239), ERK (AF0155), JNK (AF6318), p38 (AF6456), p-p65 (AF2006), p-I*κ*B*α* (AF2002), p-ERK (AF1015), p-JNK (AF3320), p-p38 (AF4001), GAPDH (AF7021), and HRP-goat anti-rabbit secondary antibody (S0001) were obtained from Affinity Biosciences (OH, USA). All other reagents and chemicals used in the study were of analytical grade.

### 2.2. Animals Experiments

Male Kunming (KM) mice (18–23 g, 4 weeks of age) were obtained from the Laboratory Animal Center of Guangzhou University of Chinese Medicine (Guangzhou, Guangdong, China) and placed in a specific pathogen-free (SPF) facility. Throughout the study, all mice were kept in a standard 12 h light/dark cycle under ambient temperature (23–25°C) and humidity (40–60%) with free access to water and a standard chow diet. All mice were fed with appropriate diet for a week before the formal experiment. All experimental procedures were conducted under the ethics approval from the Institutional Animal Care and Welfare Committee of Guangzhou University of Chinese Medicine and strictly followed the institutional guidelines (registration no. 20200715005).

### 2.3. Experimental Protocols

**Figure 1:**
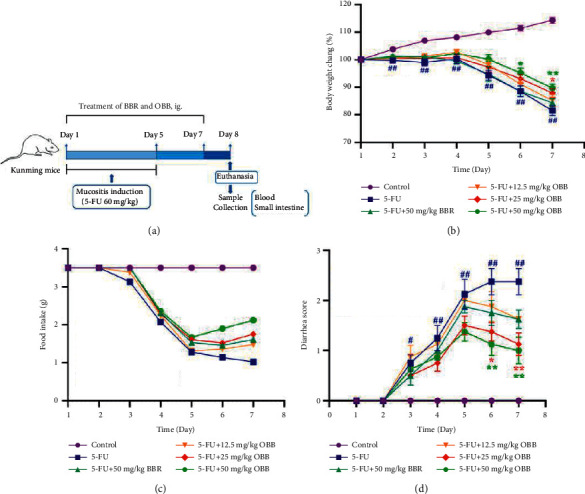
The effect of OBB on weight loss, food-intake, and diarrhea in 5-FU-induced IM mice. (a) Schematic illustration of the experiment design, (b) body weight, (c) food-intake, and (d) diarrhea score. Data were shown as mean ± SEM (*n* = 8). ^##^*P* < 0.01 vs. the control group; ^*∗*^*P* < 0.05 and ^*∗∗*^*P* < 0.01*vs.* the 5-FU group.

The experiment included six groups: the control group, 5-FU, 5-FU with BBR (BBR, 50 mg/kg, and positive control), and 5-FU with OBB (OBB, 12.5, 25, and 50 mg/kg). The dosage of 5-FU was adopted based on previous reports [19], and the doses of BBR and OBB were selected according to Li et al. [8] and our preliminary experiment. BBR and OBB were suspended in 0.5% of CMC-Na solution. Except for the control group, the other groups were given 5-FU (60 mg/kg) for 5 days once daily to establish the IM model. Simultaneously, the BBR and OBB groups were given orally 0.5 hours prior to 5-FU administration from day 1 to day 7, and mice in control and 5-FU groups were supplied with 0.5% of CMC-Na solution (Figure 1(a)). All mice were monitored and weighed daily during this period. On the 8th day, all mice were euthanized and analyzed after fasting overnight.

### 2.4. Sample Collection and Analysis

The whole blood samples were obtained from the retro-orbital and then centrifuged at 3000 rpm for 10 min at 4°C to collect the serum. 0.1 g of the small intestine tissue was fixed with 4% paraformaldehyde and examined by subsequent histology and immunohistochemistry. The remaining small intestine samples were collected and quickly preserved at −80°C refrigerator for the following analysis.

### 2.5. Assessment of Disease Activities

Throughout the experiment, body weight, food intake, and diarrhea were recorded once a day. Each mouse was given a reasonable diet according to the standard of 3.5 g/kg food. The severity of diarrhea was assessed based on the stool consistency [20]. The details are shown in Table 1.

### 2.6. Serum and Intestinal Biochemical Analysis

The concentrations of IL-8, IL-6, TNF-*α*, and IL-10 in serum were determined by ELISA kits, and the SOD level was measured by using the SOD assay kit. The intestinal tissues were homogenized with PBS and centrifuged at 14000 g for 15 min at 4°C, and then the supernatant was collected for measurement of the levels of MDA, GSH, and MPO by using commercial kits. All experiments were performed in strict accordance with the manufacturers' instructions.

### 2.7. Histopathology Evaluation

Intestinal specimens were fixed with 4% of paraformaldehyde for 24 h and embedded in paraffin. Subsequently, the specimens were cut into 3-5 *μ*m thickness and then stained with hematoxylin-eosin (H&E). Histological changes were visualized under a magnified 200 times BX53 light microscope (Olympus Co., Tokyo, Japan). The histological damage severity was assessed according to different parameters: villus height, crypt disruption, inflammatory infiltration, and integrity of the epithelium. Each parameter was assigned a score between 0 (no damage) and 3 (extreme damage). The final score was presented as the sum of each mentioned index score. The histopathological scoring criteria are based on previous descriptions with slight modification [1, 21]. The details are shown in Table 2.

### 2.8. Immunohistochemical Staining

The COX-2 expression was detected by standard immunohistochemistry techniques. The processed paraffin sections (5 *μ*m) were deparaffinized and dehydrated. The samples were treated with 3% H_2_O_2_, soaked in 10% of goat serum for 20 min, and then incubated with COX-2 primary antibody (1 : 200 dilution) at 4°C overnight. After washed with PBS, these sections were stained with HRP-conjugated secondary antibody and then washed with PBS. Finally, the slices were incubated with 3,3′-diaminobenzidine (DAB) followed by counterstaining with diluted Harris' hematoxylin and then performed by immunohistochemical staining (brown staining) under a light microscope (200x). The IOD of the slices were counted by using the Image-Pro Plus program.

### 2.9. qRT-PCR Analysis

The total RNA in the small intestine specimens was extracted using Trizol (Thermo Fisher Scientific, Waltham, MA, USA) according to the manufacturer's instructions. Then, the obtained RNA was reverse transcribed into cDNA by using the HiScript II QRT SuperMix kit (Vazyme Biotech, Nanjing, China). The mRNA expressions of iNOS, COX-2, PCNA, occludin, mucin-1, and ZO-1 were determined by using the ChamQ SYBR qPCR Master Mix kit (Vazyme Biotech, Nanjing, China). The mRNA expressions were calculated and normalized to GAPDH with the 2^−∆∆Ct^ method [21]. The primer sequences used in this assay are shown in Table 3.

### 2.10. Western Blotting Analysis

The small intestine specimens were homogenized using a RIPA lysis buffer containing protease and phosphatase inhibitors and then centrifuged at 14,000 g for 10 min at 4°C to obtain total proteins. The total proteins were denatured with a loading buffer and heated in the microwave at 100°C for 10 min. The protein samples were separated by 10% of SDS-PAGE gel, and then they were transferred to PVDF membranes. Subsequently, the membranes were blocked with 5% of defatted milk dissolved in TBST for 1 h and incubated with primary antibodies at 4°C overnight. The primary antibodies contained GAPDH, IRAK-1, I*κ*B*α*, p65, JNK, ERK, p38, p-I*κ*B*α*, p-p65, p-JNK, p-ERK, p-p38 (above antibodies were used at a dilution of 1 : 1000), MyD88 (1 : 1500), and TLR4 (1 : 2000). After washing thrice with TBST, the membranes were then reacted with HRP-conjugated secondary antibodies (1 : 3000) for 1 h. Eventually, the blots were observed using ECL detection reagents and quantified by ImageJ software. All results were normalized to the expression of GAPDH.

### 2.11. Data Analysis

Data were expressed by the mean ± standard error of mean (SEM), using a statistical analysis software SPSS 26.0 (IBM Corp., Armonk, NY, USA). The results with a normal distribution were analyzed by ANOVA (one-way analysis of variance) followed by the Dunnett's T3 test or the LSD test. The data acquired from nonparametric distribution were performed by the Mann–Whitney test. A value of *P* < 0.05 or *P* < 0.01 were recorded as statistically significant.

## 3. Results

### 3.1. The Effect of OBB on Body Weight Loss, Food-Intake, and Diarrhea

As illustrated in Figure 1(b), the body weight of mice in the control group increased continuously and steadily. The average body weight increased by 14.32 ± 3.15% of the original weight on the 7th day, while that of the 5-FU group decreased from the 5th day and decreased by 18.47 ± 5.00% of the original weight on the last day. In contrast, the body weight of mice pretreated with OBB and BBR recovered greatly in a dose-dependent manner from day 4 to day 7. The food-intake is a widely used index in chemotherapy. As revealed in Figure 1(c), the food-intake of mice in the 5-FU group decreased significantly, while OBB and BBR administration ameliorated anorexia induced by 5-FU. As shown in Figure 1(d), there was no significant diarrhea occurred in all groups on the 1st and 2nd day. The diarrhea scores continuously increased after the 5-FU treatment from day 3, and average diarrhea score was up to 2.5 on the last day. OBB dose-dependently alleviated severe diarrhea induced by 5-FU. Taken together, the results indicated that compared with the 5-FU group, administration with OBB could alleviate diarrhea and increase food-intake and body weight.

### 3.2. Effect of OBB on Histopathological Analysis and MPO Activity

**Figure 2:**
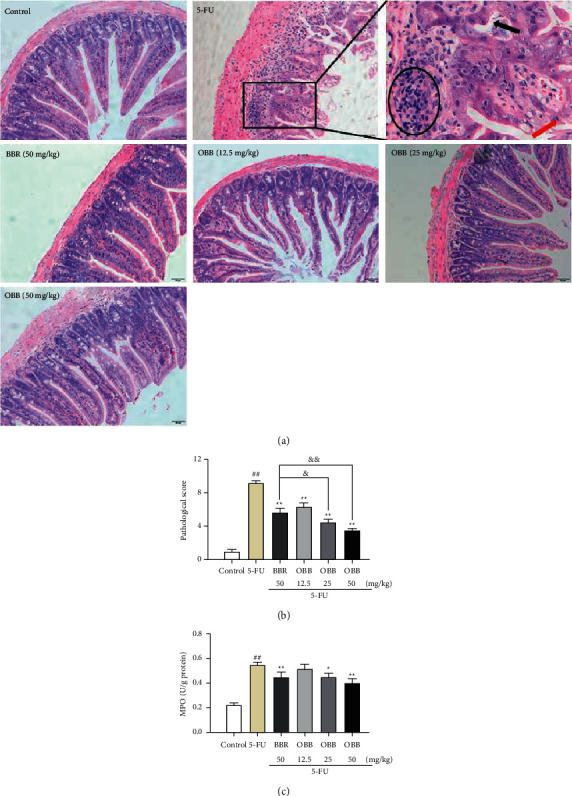
The effect of OBB on histopathological analysis and the MPO activity in 5-FU-induced IM mice. (a) Representative photographs of H&E staining (magnification ×200, the black arrow represents crypt destruction, the red arrow indicates villus degeneration, and black circle represents neutrophils infiltration). (b) Histological score (*n* = 3). (c) The MPO activity (*n* = 8). Data were presented as mean ± SEM. ^##^*P* < 0.01 vs. the control group; ^*∗*^*P* < 0.05 and ^*∗∗*^*P* < 0.01 vs. the 5-FU group; ^&^*P* < 0.05 and ^&&^*P* < 0.01*vs*. the BBR group.

Pathological changes reflect the severity of intestinal mucositis. As illustrated in Figure 2(a), the intestinal tissues in the control group sustained an optimum mucosa structure, while those of the 5-FU group mice displayed disrupted crypts, degenerative villus, inflammatory cell infiltration, and necrotic mucosa with loose epithelium, thus leading to a higher histological score than those in the control group (9.16 ± 0.75 *vs.* 1.00 ± 0.63, *P* < 0.01, Figure 2(b)). However, administration with OBB remarkably declined histological scores in a dose-dependent manner and restored the damaged intestines. It is worth noting that the therapeutic effect of OBB (25 and 50 mg/kg) was better than that of BBR (50 mg/kg).

As shown in Fig.  2(c), 5-FU administration caused a significant increase in the MPO activity. OBB (25 and 50 mg/kg) and BBR pretreatment obviously inhibited the augment of MPO (*P* < 0.05*vs*. the 5-FU group). The results showed that OBB reversed mucositis progression via weakening infiltration of neutrophil granulocytes.

### 3.3. Effect of OBB on 5-FU-Induced Oxidative Stress

**Figure 3:**
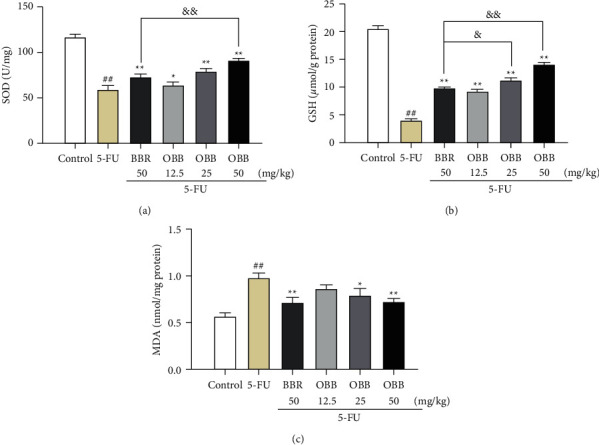
The effect of OBB on 5-FU-induced oxidative stress. (a) SOD, (b) GSH, and (c) MDA. Data were presented as mean ± SEM (*n* = 8). ^##^*P* < 0.01 vs. the control group; ^*∗*^*P* < 0.05 and ^*∗∗*^*P* < 0.01 vs. the 5-FU group; ^&^*P* < 0.05 and ^&&^*P* < 0.01*vs*. the BBR group.

To analyze effects of OBB on oxidative stress, the levels of GSH, SOD, and MDA were determined in the present study. As shown in Figure 3, the MDA level in the 5-FU group markedly elevated, while the levels of SOD and GSH were particularly lower than those in the control group mice (*P* < 0.01). However, the treatment with OBB prominently suppressed the augment of the MDA level and the decline of GSH and SOD activities dose-dependently compared with the 5-FU group (*P* < 0.05 or *P* < 0.01). Importantly, OBB (50 mg/kg) exhibited a more appreciable antioxidant effect relative to BBR at the same dose. These data showed that OBB could alleviate 5-FU-induced oxidative stress in small intestinal tissue of mice.

### 3.4. The Effect of OBB on the Expressions of COX-2 and iNOS

**Figure 4:**
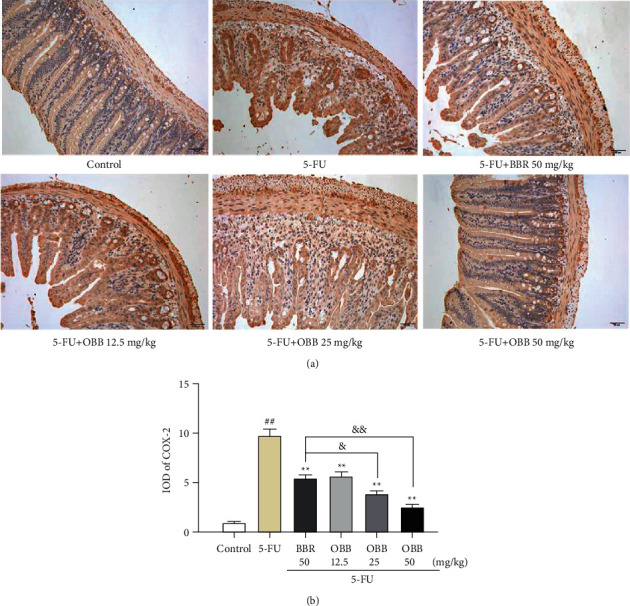
The effect of OBB on the expression of COX-2 in 5-FU-induced IM mice. (a) Representative photographs of COX-2 immunohistochemical staining (magnification ×200). (b) Integrated optical density (IOD) of COX-2 expression. The results were expressed as mean ± SEM (*n* = 3). ^##^*P* < 0.01 vs. the control group; ^*∗*^*P* < 0.05 and ^*∗∗*^*P* < 0.01 vs. the 5-FU group; ^&^*P* < 0.05 and ^&&^*P* < 0.01*vs*. the BBR group.

COX-2 plays a key role in the signal magnification stage of mucositis. Therefore, we detected the activation of COX-2 by immunohistochemistry. As revealed in Figures 4(a) and 4(b), the intensity of positive expression of COX-2 in the 5-FU group was dramatically augmented as compared to that in the control group (*P* < 0.01). On the contrary, OBB and BBR remarkably inhibited the positive expression of COX-2 (*P* < 0.01), especially OBB (25 and 50 mg/kg) exhibited a superior effect to BBR (50 mg/kg) in restraining the level of COX-2.

**Figure 5:**
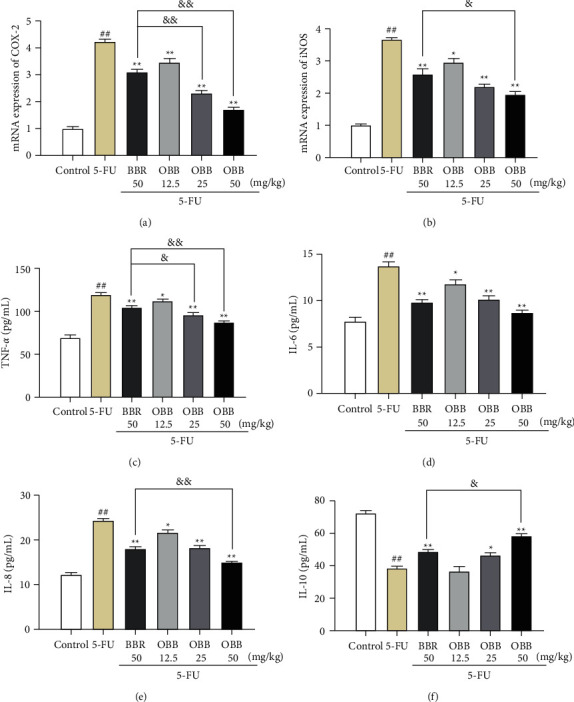
The effect of OBB on the mRNA expressions of inflammatory mediators, including COX-2 (a), iNOS (b), TNF-*α* (c), IL-6 (d), IL-8 (e) and IL-10 (f) in 5-FU-induced IM mice. The results were expressed as mean ± SEM (*n* = 6–8). ^##^*P* < 0.01 vs. the control group; ^*∗*^*P* < 0.05 and ^*∗∗*^*P* < 0.01 vs. the 5-FU group; ^&^*P* < 0.05 and ^&&^*P* < 0.01*vs*. the BBR group.

In addition, as depicted in Figures 5(a) and 5(b), after an injection of 5-FU, the mRNA expressions of COX-2 and iNOS were obviously increased compared to the control group (*P* < 0.01). Nevertheless, these increased expressions were dramatically decreased by OBB treatment (*P* < 0.01). Furthermore, compared with the BBR group, there were lower expressions of COX-2 and iNOS mRNA in intestinal tissues of the OBB group mice.

### 3.5. The Effect of OBB on the Levels of Inflammatory Cytokines

The expressions of proinflammatory and anti-inflammatory cytokines in the small intestine tissue are illustrated in Figures 5(c)–5(f). The injection of 5-FU caused a significant increase of TNF-*α*, IL-8, and IL-6, and a remarkable decline of IL-10 as compared to the control group (*P* < 0.05 or *P* < 0.01). On the contrary, the OBB treatment reversed the elevation of TNF-*α*, IL-8, and IL-6, and the decrease of IL-10 dose-dependently. The overall therapeutic effect of OBB was better than that of BBR at the same dose in 5-FU-induced IM mice. These data suggested that OBB could effectively reduce the inflammation response caused by 5-FU.

### 3.6. Gene Expression Related to Cellular Proliferation and Intestinal Barrier

**Figure 6:**
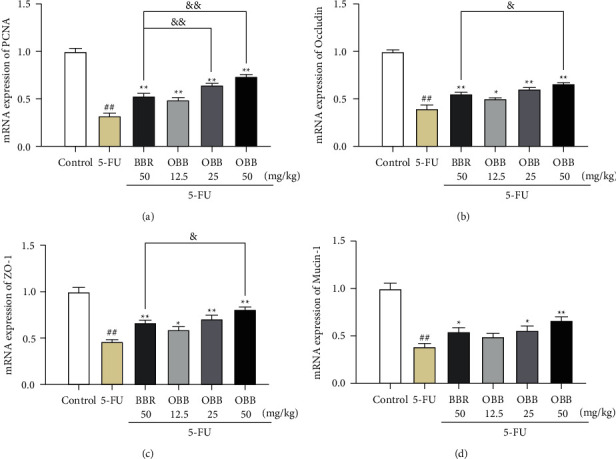
The effect of OBB on the mRNA expressions of PCNA (a), occludin (b), ZO-1 (c), and mucin-1 (d) in 5-FU-induced IM mice. The results were expressed as mean ± SEM (*n* = 6). ^##^*P* < 0.01 vs. the control group; ^*∗*^*P* < 0.05 and ^*∗∗*^*P* < 0.01 vs. the 5-FU group; ^&^*P* < 0.05 and ^&&^*P* < 0.01*vs*. the BBR group.

The activation of PCNA was detected by using qRT-PCR analysis (Figure 6(a)). As expected, the mRNA expression of PCNA was significantly higher in IM mice than that in control mice (*P* < 0.01). Nevertheless, the OBB and BBR treatment obviously alleviated the alterations in the mRNA level of PCNA induced by 5-FU (*P* < 0.01). The levels of genes associated with intestinal permeability, such as mucin-1, occludin, and ZO-1, were markedly decreased in the 5-FU group as shown in Figures 6(b)–6(d) (*P* < 0.01*vs*. the control group). However, the decrease of these genes was remarkably inhibited by OBB and BBR (*P* < 0.05 or *P* < 0.01). Moreover, OBB possessed a stronger therapeutic effect on improving intestinal permeability and proliferation than BBR at the same dosage.

### 3.7. The Effect of OBB on the TLR4/NF-*κ*B and MAPK Signaling Pathways

**Figure 7:**
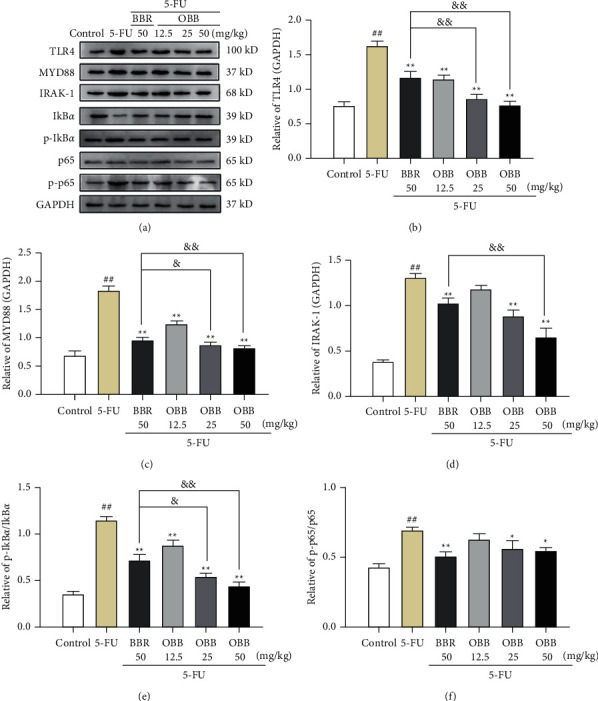
The effect of OBB on the TLR4/NF-*κ*B signaling pathway in 5-FU-induced IM mice. (a) Representative western blotting band, (b) TLR4, (c) MYD88, (d) IRAK-1, (e) the p-I*κ*B*α*/I*κ*B*α* ratio, and (f) the p-p65/p65 ratio. The results were expressed as mean ± SEM (*n* = 3). ^##^*P* < 0.01 vs. the control group; ^*∗*^*P* < 0.05 and ^*∗∗*^*P* < 0.01 vs. the 5-FU group; ^&^*P* < 0.05 and ^&&^*P* < 0.01*vs*. the BBR group.

**Figure 8:**
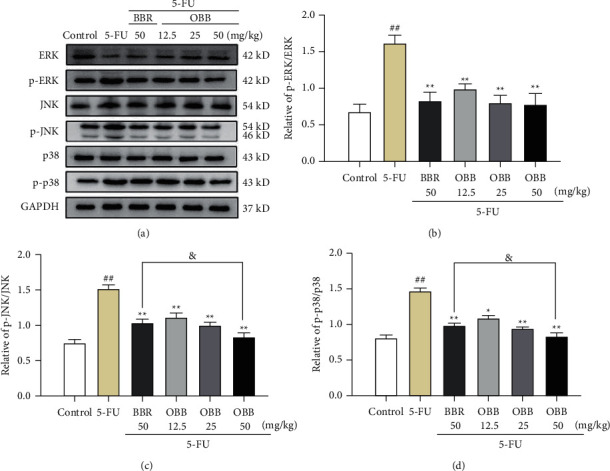
The effect of OBB on the MAPK signaling pathway in 5-FU-induced IM mice. (a) Representative western blotting band, (b) the p-ERK/ERK ratio, (c) the p-JNK/JNK ratio, and (d) the p-p38/p38 ratio. The results were expressed as mean ± SEM (*n* = 3). ^##^*P* < 0.01 vs. the control group; ^*∗*^*P* < 0.05 and ^*∗∗*^*P* < 0.01 vs. the 5-FU group; ^&^*P* < 0.05 and ^&&^*P* < 0.01*vs*. the BBR group.

Since TLR4/NF-*κ*B and MAPK signaling pathways are two prototypical inflammatory signaling pathways, we examined the inhibitory effect of OBB on 5-FU-induced MAPK and NF-*κ*B pathways. As seen in Figures 7 and 8, the protein expressions of TLR4, MyD88, IRAK-1, p-p65, p-I*κ*B*α*, p-ERK, p-JNK, and p-p38 were elevated sharply after 5-FU injection as compared with those in the control group mice (*P* < 0.01). In contrast, these protein expressions were, especially, inhibited by pretreatment with OBB and BBR (all *P* < 0.05, *vs*. the 5-FU group). Particularly, OBB (50 mg/kg) exhibited a superior effect on the TLR4/NF-*κ*B signaling pathway to BBR at the same dosage. The data indicated that OBB alleviated the inflammatory response of intestinal mucosal tissue through the TLR4/NF-*κ*B and MAPK signaling pathways.

## 4. Discussion

BBR has been considered as a promising drug candidate for treating intestinal mucositis [9], although it possesses an extremely low bioavailability. Pharmacokinetic studies have indicated that BBR undergoes extensive metabolism, resulting in a low plasma concentration. Therefore, many researchers have focused their attention on metabolites of BBR [13, 22]. A previous study has suggested that, about 43.5% of BBR is absorbed in the gastrointestinal tract, suggesting that intestinal flora played a key role in BBR metabolism [23]. Our previous research found that the intestinal microflora could convert BBR into OBB by oxidation reaction [8]. In particular, the C-8 quaternary ammonium structure of BBR is transformed into a lactam ring with stronger lipophilic properties, which could significantly enhance the biological activity of BBR. With improved biocompatibility and more favorable safety, OBB exerted superior anti-inflammatory [15], antifungal [16], antiarrhythmic [17], and hypoglycemic [18] bioactivities compared with BBR. In this study, our results showed that, under the same dosage, OBB had a better efficacy than BBR in 5-FU-induced IM mice. The protective effect of OBB may be due to maintenance of intestinal barrier integrity, inhibition of the TLR4-linked NF-*κ*B and MAPK signaling pathways, and subsequent downregulation of oxidative stress and inflammatory mediators.

In this study, a typical mucositis mouse model induced by 5-FU was established, and its clinical symptoms were similar to those of human IM. Weight loss, diarrhea, and anorexia are common side effects of 5-FU chemotherapy, which are considered as important parameters to evaluate the severity of mucositis [24]. Our results showed 5-FU promoted weight loss, inappetence, and diarrhea, which was consistent with the previous study [25]. The OBB treatment could markedly ameliorate these clinical symptoms, showing a better efficacy than BBR. Histopathological evaluation indicated that OBB restored the 5-FU-elicited intestinal structure damage such as villi degeneration, crypt necrosis, epithelial loosening, and inflammatory cell infiltration, which suggested that OBB could promote mucosal healing during chemotherapy. MPO, a key enzyme secreted by neutrophils, is considered an indicator of the degree of inflammatory infiltration [26]. 5-FU injections could cause neutrophils to release excessive MPO, promote inflammatory process, and exacerbate tissue damage [27]. In this study, OBB prevented 5-FU-induced MPO release dose-dependently, which was consistent with the results in histopathological examination.

Intestinal barrier dysfunction is a significant feature of IM. Intestinal inflammation, slow gene proliferation, and permeability changes can cause intestinal barrier dysfunction, which could cause a vicious circle [28–30]. The mucosal barrier is the first line of protection of the intestinal tract, which is composed of epithelial cells, mucous layer, and intercellular junctions. Tight junctions (TJs) are the apical intercellular structures in epithelial cells, which regulate the cell-to-cell adhesion, epithelial permeability, and paracellular movement. TJs are composed of variety of proteins, such as claudins, occludin, and peripheral membrane protein ZO (ZO-1 and ZO-2) [31]. A decrease of TJs could lead to the increase of mucosal permeability and the injury of the mucosal barrier [32]. The main component of the mucus layer is mucins like mucin-1 secreted by goblet cells of intestinal epithelium, whose function is to protect the intestinal tract from the invasion of pathogens [33]. The damage of intestinal epithelial mucin-1 could cause the increase of intestinal permeability, allowing bacteria and toxins to easily penetrate the damage mucosal barrier, and lead to the occurrence and development of IM [34, 35]. Hence, enhancing the function of the intestinal mucosal barrier may be a potential therapeutic target to alleviate intestinal inflammation [33]. Previous findings suggested that BBR and its metabolites could restore intestinal barrier dysfunction via upregulating the expressions of TJs and mucins in 5-FU treated mice [8–10]. In this study, we detected that the mRNA expressions of mucin (mucin-1) and TJ (occludin and ZO-1) were markedly decreased after 5-FU injection. Nevertheless, OBB reversed the downregulation of mucin-1, occludin and, ZO-1 caused by 5-FU. In addition, the function of the intestinal barrier also depends on the intensity physiological renewal [36]. Proliferating cell nuclear antigens (PCNA) is an auxiliary protein of DNA polymerases, which reflects directly the proliferation of cells [5]. It is reported that 5-FU interfered with DNA synthesis by inhibiting thymidylate synthase, which directly slowed the proliferation of intestinal epithelial cells in IM mice [9]. Our results indicated that OBB effectively increased the expression of PCNA and then maintained the numerous functional activities of intestinal mucosal epithelium. According to these results, we speculated that OBB had an obvious protective effect on the destruction of intestinal mucosal barrier induced by 5-FU.

Oxidative stress is an important mechanism in the initiation of IM [37, 38]. 5-FU has been reported to cause IM by generating reactive oxygen species (ROS). Excessive ROS produce oxidative stress on cells, destroy protein, DNA, lipid membranes, and other cellular components, and ultimately induce cytotoxicity [39, 40]. Thus, scavenging free radicals and enhancing enzymatic antioxidant defense system are important mechanisms to protect against 5-FU-induced IM mice. As a chief enzymatic antioxidant, GSH protects the body from oxidative damage by scavenging free radicals. Conversely, GSH deficiency may cause ROS to seriously attack antioxidants such as SOD, thus destroying cellular antioxidant defense mechanism [41]. A decrease of antioxidant enzymes leads to lipid peroxidation, which were indicated by a high MDA level [19]. The existing study reported that 5-FU injection could trigger the oxidative stress by inhibiting the expressions of GSH and SOD, and elevating the level of MDA [39]. Interestingly, the OBB and BBR pretreatment could reverse the increase of MDA level and prevent the consumption of antioxidant enzymes in 5-FU-induced IM mice. Moreover, the antioxidant effect of OBB was better than that of BBR. The results suggested that OBB could improve 5-FU-induced IM mice by restoring antioxidant defense and preventing lipid peroxidation.

Inflammatory cytokines play a central role in the development of IM, which is correlated to the activation of NF-*κ*B and MAPK pathways [6]. The balance of proinflammatory/anti-inflammatory factors is the basis of maintaining intestinal homeostasis and protecting the mucosa from damage [42]. Among them, proinflammatory cytokines such as IL-8, IL-6, and TNF-*α* could facilitate the immune response by augmenting the recruitment of leukocytes and neutrophils, promoting secretion of other cytokines, and cell-cell signal transduction [7]. Contrarily, anti-inflammatory mediators including IL-10 could attenuate the symptoms of mucositis and inhibit its progression by limiting secretion of proinflammatory cytokines [43]. Thus, regulating the balance of these inflammatory cytokines is essential for the treatment of IM. However, the balance of these mediators was destroyed in 5-FU-induced mice [5]. Our results indicated that the OBB pretreatment reduced the levels of TNF-*α*, IL-8, and IL-6 and raised the IL-10 level. These results implicated that the OBB pretreatment alleviated 5-FU-induced IM mice through regulating inflammatory cytokine levels. NO and PGE2 are recognized inflammatory markers, which can be dominated by TNF-*α* [44]. Overproduction of NO can trigger inflammation and enhance the activity of COX-2 [19]. As a cyclooxygenase to product PGE2, COX-2 is only detectable in certain pathophysiological conditions including inflammation, malignant transformation, and tissue damage. Studies have shown that 5-FU can cause the decrease of COX-2 and iNOS [39]. In our present study, OBB administration significantly downregulated the gene expressions of COX-2 and iNOS induced by 5-FU in a dose-dependent manner. Immunohistochemical analysis of COX-2 further supported the preventive potential of OBB on 5-FU-induced IM mice. Particularly, OBB exhibited superior anti-inflammatory capacity to BBR. Taken together, the anti-inflammatory effect of OBB on 5-FU-induced IM mice may be closely related to alleviating inflammatory state by regulating inflammatory related factors.

It is reported that inflammatory response in the intestinal mucosa is the pathophysiology of IM [21]. Indeed, TLRs play a key role in the initiation of inflammatory response by activating the MyD88-dependent and independent pathways. The activation of MyD88 could be triggered by overproducing toll-like receptor 4 (TLR4), which is an essential adaptor protein of TLR signaling pathway. And then MyD88 combined with IRAK-1 activates the IRAK-1, which in turn initiates NF-*κ*B and MAPK phosphorylation [45]. The activation of NF-*κ*B and MAPK increased the release of numerous inflammatory cytokines. Meanwhile, the interaction of inflammatory factors can promote the activation of the MAPK and NF-*κ*B signaling pathways, further amplifying the inflammatory response, and eventually causing inflammatory injury. In this study, the pretreatment of OBB decreased the mRNA and protein expressions of TLR4, MyD88, and IRAK-1, which prompts that the anti-inflammatory effect of OBB, and might be closely related to the downregulation of the TLR4/MyD88 pathway.

**Figure 9:**
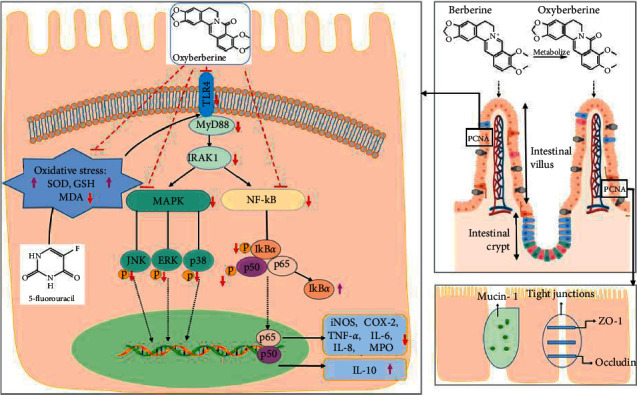
Summary scheme of the protective mechanisms of OBB on 5-FU-induced IM mice.

NF-*κ*B is a transcription factor of Rel protein family that regulates the expression of various inflammatory genes. Under normal conditions, NF-*κ*B is localized in cytoplasm. Once oxidative stress triggers an inflammatory response, NF-*κ*B is activated and then translocated into nucleus, stimulating the release of downstream genes (I*κ*B*α* and p65) [40]. In our finding, OBB significantly inhibited the NF-*κ*B pathway by inhibiting I*κ*B*α* and p65 phosphorylation and translocation of p65. The MAPK pathway has been reported to enhance the activity of NF-*κ*B by a complex mechanism. ERK, JNK, and p38 are considered to be the main kinases in the MAPK pathway that implicated in inflammation, apoptosis, neurogenesis, and many signal transduction [46]. After phosphorylation, the proteins interconnected with the MAPK pathway are activated and then regulated the downstream targets including NF-*κ*B, TNF-*α*, and so on, thus controlling inflammation [47]. In our research, the OBB pretreatment was found to remarkably inhibit the phosphorylation of ERK, JNK, and p38 in 5-FU-induced IM model. Particularly, the effects of OBB (25 mg/kg and 50 kg/mg) were superior to BBR on inhibiting NF-*κ*B and MAPK pathway phosphorylation. Taken together, our results revealed that the anti-inflammatory effect of OBB on 5-FU-induced IM mice might be intimately linked to the constraint of the TLR4/MyD88-mediated NF-*κ*B and MAPK pathways (Figure 9).

## 5. Conclusion

In conclusion, the current results showed that OBB protected against 5-FU-induced IM mice by significantly suppressing the inflammatory disturbances, restoring intestinal barrier function, improving injury of intestinal mucosa, and restricting the clinical symptoms. Its protective effect may be related to maintaining the integrity of intestinal barrier, suppressing of the TLR4-linked NF-*κ*B and MAPK signaling pathway and subsequent downregulating of inflammatory mediators. It was noteworthy that the therapeutic effect of OBB was superior to BBR. Therefore, it is believed that OBB might be the main bioactive form of antimucositis in BBR in *in vivo*. OBB might be an effective therapeutic drug to alleviate the adverse reactions of 5-FU chemotherapy. Further effort should be carried out to explore its efficacy and potential mechanism in other experimental models mimicking intestinal mucositis.

## Figures and Tables

**Table 1:** Diarrhea score.

Score	Diarrhea extent
0	Normal: normal stool or absent
1	Mild: slightly wet and soft stool
2	Moderate: wet and unformed stool with moderate perianal staining of the coat
3	Severe: watery stool with severe perianal staining of the coat

**Table 2:** Histopathological score.

Score	Inflammation extent	Crypt aberrant	Villus damage	Integrity of the epithelium
0	None	Intact crypt	Intact villus	Intact epithelium
1	Mild	Loss of basal 1/3 of crypt	Shortening of height 1/3 of villus	Loss of 1/3 of epithelium
2	Moderate	Loss of basal 2/3 of crypt	Shortening of height 2/3 of villus	Loss of 2/3 of epithelium
3	Severe	Loss of entire crypt	Degeneration of entire villus	Loss of entire epithelium

**Table 3:** Sequences of primers used for qRT-PCR.

Gene	Forward primer (5′ to 3′)	Reverse primer (3′ to 5′)
COX-2	GAAGATTCCCTCCGGTGTTT	CCCTTCTCACTGGCTTATGTAG
iNOS	ACTCAGCCAAGCCCTCACCTAC	TCCAATCTCTGCCTATCCGTCTCG
PCNA	TGAAGAAGGTGCTGGAGGCTCTC	AGCTGTACCAAGGAGACGTGAGAC
Occludin	TGGCTATGGAGGCGGCTATGG	AAGGAAGCGATGAAGCAGAAGGC
Mucin-1	CATTCCAGACCACAATGGCTCCTC	ATTGACTTGGCACTGAAGGCTGAG
ZO-1	CCACCTCGCACGCATCACAG	TGGTCCTTCACCTCTGAGCACTAC
GAPDH	GCACAGTCAAGGCCGAGAATGG	GGTGGCAGTGATGGCATGGAC

## Data Availability

The data used to support the findings of this study are available from the corresponding author upon request.

## Abbreviations

5-FU: 5-Fluorouracil
BBR: Berberine
COX-2: Cyclooxygenase-2
ERK: Extracellular signal-regulated kinase
GAPDH: Glyceraldehyde-3-phosphate dehydrogenase
GSH: Glutathione
H&E: Hematoxylin and eosin
IM: Intestinal mucositis
OBB: Oxyberberine
iNOS: Inducible nitric oxide synthase
IL-6: Interleukin-6
IL-8: Interleukin-8
IL-10: Interleukin-10
I*κ*B*α*: IkappaB-alpha
IRAK-1: Interleukin-1 receptor-associated kinase 1
JNK: c-Jun N-terminal kinase
MAPK: Mitogen-activated protein kinase
MDA: Malondialdehyde
MPO: Myeloperoxidase
MyD88: Myeloid diﬀerentiation factor 88
NF-*κ*B: Nuclear factor-kappa B
p-I*κ*B*α*: PhosphoIkappaB alpha
p-ERK: Phospho-extracellular signal-regulated kinase
p-JNK: Phospho-c-jun N-terminal kinase
ROS: Reactive oxygen species
SOD: Superoxide dismutase
TLR4: Toll-like receptor 4
TNF-*α*: Tumor necrosis factor alpha
ZO-1: Zonula occludens 1.
